# Metastasis of Squamous Cell Carcinoma of the Larynx to the Right Adrenal Gland—A Case Report

**DOI:** 10.3390/clinpract15030049

**Published:** 2025-02-26

**Authors:** Eliza Działach, Michał Simlot, Karolina Osowiecka, Elżbieta Nowara, Jarosław Markowski, Mateusz Grajek

**Affiliations:** 1 Department of Public Health, Faculty of Public Health in Bytom, Medical University of Silesia in Katowice, Piekarska 18, 41-902 Bytom, Poland; 2 Department of Otorhinolarygology, Faculty of Medicine in Katowice, Medical University of Silesia in Katowice, Francuska 20-24, 40-027 Katowice, Poland; msimlot@sum.edu.pl (M.S.); jmarkowski@sum.edu.pl (J.M.); 3 Department of Psychology and Sociology of Health and Public Health, School of Public Health, University of Warmia and Mazury in Olsztyn, Warszawska 30, 10-082 Olsztyn, Poland; karolina.osowiecka@uwm.edu.pl; 4 Department of Health Science, Jan Długosz University of Humanities and Natural Sciences, Waszyngtona 4/8, 42-200 Częstochowa, Poland; elizabieta.nowara@interia.pl

**Keywords:** squamous cell carcinoma, adrenal metastasis, PET-CT imaging, interdisciplinary approach, rare malignancy

## Abstract

**Background/Objectives**: Malignant adrenal tumors comprise both primary adrenal neoplasms and metastatic lesions, with the latter being significantly more common. Squamous cell carcinoma (SCC) of the larynx is a prevalent head and neck cancer that typically spreads to the cervical lymph nodes, with distant metastases being rare. Among such metastases, adrenal gland involvement is particularly uncommon, presenting unique diagnostic and therapeutic challenges. The study aimed to explore the progression, diagnostic process, and therapeutic management of a rare case of SCC of the larynx metastasizing to the adrenal gland, highlighting the role of advanced diagnostic imaging and a multidisciplinary approach in patient care. **Methodology**: A 66-year-old male with grade 3 SCC of the larynx underwent total laryngectomy, selective cervical lymphadenectomy, and radiotherapy with a dose of 70 Gy. Chemotherapy was discontinued due to hematological complications. Post-treatment monitoring included CT and PET-CT imaging, leading to the detection of a large adrenal mass. Surgical biopsy confirmed metastatic SCC in the adrenal gland, as resection was not feasible due to extensive invasion. Diagnostic imaging and histopathological examination were complemented by biochemical evaluations to assess hormonal activity. **Results**: The adrenal mass was identified as a metastasis from the laryngeal SCC. Imaging studies provided detailed insights into the lesion’s size, metabolic activity, and non-functional status. Despite comprehensive efforts, the tumor was deemed unresectable, highlighting the aggressive nature of the disease and the limitations of current therapeutic modalities. **Conclusions**: This rare case emphasizes the importance of early detection, advanced imaging techniques, and interdisciplinary collaboration in managing complex metastatic presentations. It underscores the critical need for further research into systemic treatments, such as immunotherapy, and the development of standardized protocols for rare metastatic patterns. The study contributes to the growing body of literature on the management of uncommon cancer metastases, advocating for individualized patient care and innovation in treatment strategies.

## 1. Background

Malignant adrenal tumors represent a heterogeneous group of pathological changes encompassing primary tumors of the adrenal cortex and medulla, as well as metastatic lesions originating from other organs. This group includes primary adrenal tumors such as adrenocortical carcinoma (ACC) and medullary adrenal tumors, primarily pheochromocytoma and neuroblastoma. However, metastatic lesions in the adrenal glands are significantly more frequent than primary tumors, further complicating diagnosis and treatment [1,2].

Primary adrenal tumors often develop within the context of specific genetic syndromes. Among these syndromes, multiple endocrine neoplasia syndromes type I (MEN I), IIa (MEN IIa), and IIb (MEN IIb) are particularly notable. MEN I syndrome is characterized by tumors in the pancreas, pituitary, and parathyroid glands, whereas MEN IIa and IIb involve conditions such as medullary thyroid carcinoma, pheochromocytoma, and parathyroid hyperplasia. The inheritance of mutations in the RET gene in MEN IIa and IIb and mutations in the MEN1 gene in MEN I significantly increases the risk of adrenal tumor development [3,4].

In the general population, metastatic lesions in the adrenal glands account for up to 20–30% of all tumors in this location, making them much more common than primary adrenal tumors. Metastases to the adrenal glands most frequently originate from organs such as the lungs, kidneys, breasts, or skin (in cases of melanoma). These cancers exhibit a high propensity for hematogenous spread, explaining the frequent involvement of the adrenal glands [5,6].

Squamous cell carcinoma of the larynx is one of the most common head and neck cancers. The etiology of this disease is strongly linked to exposure to risk factors such as long-term tobacco use and excessive alcohol consumption. This disease most commonly affects men over the age of 60, likely due to prolonged exposure to carcinogens in this demographic group [7].

The spread of squamous cell carcinoma of the larynx usually involves cervical lymph nodes, with distant metastases being relatively rare. The lungs are the most common site of distant metastases, whereas adrenal metastases from this cancer are exceptionally rare, making them an intriguing clinical challenge [8,9].

Diagnostic imaging plays a pivotal role in assessing cancer staging. Methods such as computed tomography (CT) and positron emission tomography with fluorodeoxyglucose ([18F]FDG PET-CT) are widely used for early detection and precise localization of metastatic lesions. CT enables evaluation of the size, location, and characteristics of adrenal lesions, whereas PET-CT assesses the metabolic activity of tumors, aiding in distinguishing malignant from benign changes [10,11].

In recent years, advanced imaging techniques such as PET-CT using [18F]FDG have gained prominence in the diagnosis and monitoring of cancer progression, including rare metastatic squamous cell carcinoma (SCC). PET-CT not only allows for the detection of lesions with high metabolic activity but also the quantitative analysis of glucose uptake, which is crucial in assessing tumor aggressiveness and response to treatment [12,13,14]. In terms of systemic therapies, immunotherapies are growing in importance, especially PD-1/PD-L1 checkpoint inhibitors, which are showing promising results in the treatment of advanced SCC. Advances in precision medicine are also enabling the identification of predictive biomarkers that can aid in the selection of targeted therapies, increasing treatment efficacy in selected patient subgroups [12,15].

## 2. Methodology

The study employed a meticulously structured methodology to investigate the progression and treatment outcomes of squamous cell carcinoma (SCC) of the larynx, characterized by an unusual metastatic presentation in the adrenal gland. The clinical process began with an initial diagnosis involving a comprehensive clinical examination, laryngoscopic evaluation, and a histopathological biopsy, which confirmed the presence of G3 SCC of the larynx. Following this, a series of therapeutic interventions were undertaken, including a total laryngectomy, selective cervical lymphadenectomy, and tracheostomy. Postoperative care was meticulously managed to ensure optimal wound healing and patient recovery.

Subsequent oncological treatment involved a multimodal approach, combining radiotherapy at a dosage of 70 Gy and chemotherapy. Throughout this phase, the patient was closely monitored to manage complications, particularly hematological side effects. A detailed follow-up protocol was implemented, incorporating imaging studies such as CT and PET-CT scans to detect any signs of recurrence or metastatic spread. Biochemical evaluations were also conducted to rule out hormonal activity associated with the adrenal tumor.

To assess the nature of the adrenal mass, a surgical evaluation was performed through laparotomy, which allowed for an assessment of the mass’s resectability. This procedure was complemented by a surgical biopsy, providing histopathological confirmation of the tumor’s metastatic origin.

CT imaging offered a detailed anatomical evaluation of the adrenal mass and its surrounding structures. PET-CT using [18F]FDG provided a metabolic characterization of the tumor, aiding in differentiating between malignant and benign lesions and identifying potential systemic spread. These imaging findings were corroborated by a histopathological analysis, which confirmed the metastatic nature of the adrenal tumor, while biochemical tests verified its hormonally inactive status.

PET-CT (Celestion CMS, USA (Used in Katowice, Poland)) was performed using a state-of-the-art scanner, following the standard protocol for oncology FDG-PET studies. The patient received 370 MBq of [18F]FDG intravenously, followed by image acquisition 60 min after administration of the radiopharmaceutical. The maximum SUVmax uptake value of the adrenal lesion was 12.8, indicating high metabolic activity, characteristic of metastatic lesions.

Biopsy was performed under CT-guided biopsy, using fine-needle aspiration (FNA) and thick-needle biopsy (core biopsy) to obtain sufficient material for histopathological and immunohistochemical analysis. The results showed the presence of p40 and CK5/6 expression, confirming the tumor’s origin from primary laryngeal SCC.

The study adhered to rigorous ethical standards to safeguard the patient’s welfare and uphold research integrity. Informed consent was obtained in writing, with the patient agreeing to all diagnostic and therapeutic procedures as well as the use of his medical data for research purposes. To ensure patient confidentiality, all personal and medical information was anonymized. The study complied with the principles outlined in the Declaration of Helsinki and institutional ethical review board policies, ensuring that all procedures met established ethical standards.

A careful risk-benefit analysis was conducted to weigh the advantages of surgical interventions and imaging studies against the inherent risks, particularly given the advanced stage and metastatic nature of the tumor. Ethical deliberations involved an interdisciplinary team of oncologists, surgeons, radiologists, and pathologists, ensuring that all decisions prioritized comprehensive care and the patient’s best interest. This collaborative approach exemplified commitment to ethical and effective clinical management, underscoring the complexity and importance of managing such a rare and challenging presentation of squamous cell carcinoma.

## 3. Protocol

### 3.1. Study Design

The study was conducted as a single-case design, aligning with Yin’s principles of case study research [16]. The design was meticulously planned to provide an in-depth understanding of the progression, diagnostic challenges, and therapeutic outcomes associated with squamous cell carcinoma (SCC) of the larynx exhibiting rare adrenal metastasis. This study adhered to the PRISMA framework for case reports to ensure systematic reporting and reproducibility of the findings [17].

### 3.2. Research Questions

The primary research questions focused on understanding the clinical and radiological progression of squamous cell carcinoma (SCC) of the larynx with adrenal metastasis, exploring the therapeutic challenges and outcomes associated with this rare presentation and evaluating the effectiveness of multimodal interventions in managing such a case.

### 3.3. Case Selection

The subject was selected based on the rare clinical presentation of SCC of the larynx with adrenal metastasis. The inclusion criteria were a histopathologically confirmed diagnosis of G3 SCC of the larynx and evidence of adrenal metastasis confirmed through advanced imaging and a histopathological analysis.

### 3.4. Data Collection Methods

To ensure methodological rigor, data were collected using multiple sources, including clinical records, which provided detailed documentation of diagnostic and therapeutic processes; histopathology reports, confirming the primary tumor and metastatic lesion through biopsy results; advanced imaging, such as CT scans for anatomical assessment and PET-CT with [18F]FDG for metabolic evaluation and systemic spread analysis; biochemical tests to evaluate hormonal activity associated with the adrenal tumor; and intraoperative observations, including notes from laparotomy and biopsy procedures.

### 3.5. Intervention Protocol

The initial diagnosis was confirmed through a comprehensive clinical examination, laryngoscopic evaluation, and histopathological biopsy, which identified G3 squamous cell carcinoma (SCC) of the larynx. Surgical interventions included total laryngectomy, selective cervical lymphadenectomy, and tracheostomy, with postoperative care focused on ensuring optimal wound healing and recovery. Oncological treatment involved radiotherapy at a dosage of 70 Gy, along with chemotherapy, while closely monitoring for hematological side effects. The follow-up protocol consisted of imaging studies, including CT and PET-CT scans, as well as biochemical evaluations to detect any recurrence or further metastasis.

### 3.6. Data Analysis

The study included a quantitative assessment of treatment outcomes, such as tumor size reduction and recurrence rates, alongside qualitative insights into the clinical challenges and decision-making processes encountered during treatment.

### 3.7. Ethical Considerations

Informed consent was obtained from the patient for all diagnostic, therapeutic, and research-related procedures. However, due to the article being based on planned medical procedures conducted during the patient’s diagnostic and therapeutic process, ethical approval for separate consent was not required. Confidentiality was maintained by anonymizing patient data in accordance with the Declaration of Helsinki and institutional ethical policies. An interdisciplinary team conducted a detailed risk-benefit analysis, carefully weighing the potential benefits of treatment against the associated risks.

### 3.8. Multidisciplinary Collaboration

The case was discussed by a multidisciplinary team (MDT) that included oncologists, surgeons, radiologists, and pathologists. After surgical treatment, the MDT recommended radiotherapy as an adjunct treatment and planned chemotherapy, but this was discontinued due to hematological complications. After the adrenal metastasis was detected, the MDT re-evaluated the available treatment options. Due to extensive infiltration and the inability to surgically resect, it was decided to implement palliative systemic therapy. After considering the patient’s profile and previous chemotherapy-related complications, the MDT decided on PD-1 inhibitor-based immunotherapy as the optimal therapeutic option.

### 3.9. Limitations and Generalizability

This study represents a single-case design, which limits generalizability. However, the detailed documentation and systematic methodology provide valuable insights for similar rare cases and future research.

### 3.10. Reporting Standards

The study adhered to PRISMA guidelines for case reports to ensure transparency and reproducibility. All findings, including diagnostic challenges, therapeutic interventions, and outcomes, were systematically documented and reported.

## 4. Case Description and Results

The described case concerns a 66-year-old patient with a squamous cell carcinoma of the larynx metastasizing to the right adrenal gland. Such cases are extremely rare, underscoring the importance of an interdisciplinary approach to diagnosis and treatment. The precise identification of the metastasis using imaging techniques such as PET-CT allowed for optimization of the therapeutic plan, potentially improving treatment outcomes.

In April 2020, a 66-year-old male patient presented to the Department of Otorhinolaryngology at the Independent Clinical Hospital in Katowice with a diagnosis of grade 3 squamous cell carcinoma (G3 SCC) of the larynx, confirmed via biopsy. Clinical evaluation revealed advanced locoregional involvement of the tumor.

On the day of admission, the patient underwent total laryngectomy, selective cervical lymphadenectomy, and tracheostomy. The surgical procedure and perioperative period proceeded without complications, and the surgical wound healed uneventfully. After 11 days, the patient was discharged in good general and local condition.

Given the clinical stage of the malignancy, the patient was qualified for combined modality treatment, including radiotherapy with a total dose of 70 Gy and concurrent chemotherapy. Treatment commenced in June 2020. However, during therapy, hematological complications such as leukopenia and thrombocytopenia necessitated the cessation of chemotherapy. Despite these challenges, radiotherapy was completed as planned.

Post-treatment follow-up involved oncological surveillance. Imaging studies performed during the follow-up period revealed no evidence of tumor recurrence in the postoperative site.

**Figure 1:**
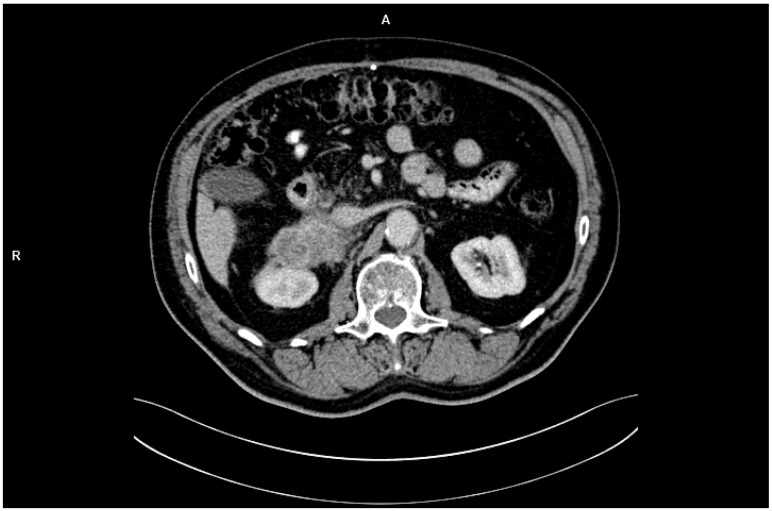
CT imaging with identified a 7 × 9 cm mass in the right adrenal gland.

In February 2023, the patient independently underwent abdominal CT imaging, which identified a 7 × 9 cm mass in the right adrenal gland, raising suspicion of metastatic disease (Figure 1). Subsequent PET-CT imaging confirmed the presence of a metabolically active tumor with high glucose uptake and additional lymphadenopathy in the retroperitoneal space. Biochemical analyses demonstrated that the adrenal tumor was hormonally inactive.

PET-CT showed intense [18F]FDG uptake in the adrenal tumor area, with a maximum SUVmax = 12.8. The elevated metabolic activity of the lesion was consistent with the tumor phenotype, while the lack of significant uptake in other organs suggested limited disease progression at the stage of detection. SUV values in adrenal adenomas typically do not exceed 3.5, which further supported the diagnosis of a metastatic lesion. The CT image showed an irregularly limited lesion measuring 7 × 9 cm, infiltrating adjacent structures. In the clinical context, these imaging features indicated the advanced nature of the metastasis and its unresectability.

In March 2023, the patient was scheduled for laparotomy with the aim of tumor resection. During surgery, the adrenal mass was deemed unresectable due to extensive invasion into surrounding structures. A surgical biopsy was performed, and histopathological examination confirmed the metastasizing of the squamous cell carcinoma of the larynx into the right adrenal gland.

A histopathological examination of the adrenal lesion revealed squamous cell carcinoma with a morphology consistent with primary laryngeal cancer. Immunohistochemistry revealed the expression of p40 and CK5/6 markers, which are characteristic of squamous cell carcinoma and consistent with the immunohistochemical profile of the primary tumor. In addition, analysis of the histological architecture confirmed the high similarity to previously diagnosed SCC of the larynx. Extensive infiltration of the tumor into the inferior vena cava and surrounding lymph nodes was confirmed, indicating inoperability. The laparoscopic examination provided important information that was not fully visible on PET-CT, including the degree of infiltration of surrounding structures and the extent of the tumor process.

This case highlights the rare occurrence of adrenal metastasis originating from squamous cell carcinoma of the larynx. Such cases are exceedingly uncommon and pose significant diagnostic and therapeutic challenges. Advanced imaging modalities, including PET-CT, played a pivotal role in identifying the metastatic lesion and evaluating its metabolic activity. The determination of the lesion’s hormonal inactivity was crucial for tailoring the treatment strategy.

The inability to achieve surgical resection underscores the aggressive nature of metastatic SCC and the importance of early detection and comprehensive management. The case also emphasizes the necessity for interdisciplinary collaboration, including oncologists, surgeons, radiologists, and pathologists, in addressing complex metastatic patterns.

Due to the progression of the disease and the lack of options for further treatment, the patient qualified for palliative systemic therapy. Due to previous hematologic complications associated with chemotherapy, the MDT decided to use immunotherapy with a PD-1 inhibitor, a choice based on the potential benefits of an immune checkpoint blockade in the treatment of advanced squamous cell carcinoma (Table 1).

## 5. Discussion

The case of a metastatic squamous cell carcinoma of the larynx spreading to the adrenal gland is exceedingly rare and poses a significant clinical challenge, both diagnostically and therapeutically. According to the current literature, metastases from laryngeal carcinoma most commonly involve cervical lymph nodes, with distant metastases—such as those to the adrenal glands—being extraordinarily uncommon [18,19]. This rarity underscores the importance of heightened clinical suspicion and thorough diagnostic evaluation in identifying such atypical presentations.

Advanced diagnostic imaging modalities are crucial in the evaluation of rare metastatic patterns. In this case, PET-CT imaging played a pivotal role by enabling precise localization of metastatic lesions and the assessment of their metabolic activity. Such imaging is invaluable not only for determining the extent of disease spread but also for differentiating between primary adrenal tumors and secondary metastatic lesions. This differentiation has critical implications for treatment planning and patient prognosis [11].

In addition to imaging, biochemical evaluations provide an essential complement in the diagnostic process. As demonstrated in this case, biochemical assays confirmed the non-functional nature of the adrenal tumor. Distinguishing between functional and non-functional adrenal tumors is a critical step in guiding clinical management, as functional adrenal tumors may require additional endocrine-specific interventions to address hormone-related symptoms or complications [20].

The management of adrenal metastases, particularly those originating from squamous cell carcinoma of the larynx, presents significant challenges. Surgical resection is typically considered the gold standard for adrenal metastases; however, this approach is feasible only in cases where complete removal of the tumor can be achieved. Unfortunately, in this case, the extent of local invasion rendered the tumor unresectable. This limitation necessitated the exploration of alternative therapeutic modalities [21,22].

Systemic treatments, such as chemotherapy, immunotherapy, and targeted therapies, may be considered as alternative options. However, their efficacy in the context of adrenal metastases from laryngeal carcinoma remains limited and under-researched. The scarcity of documented cases and corresponding clinical trials poses a significant barrier to establishing standardized treatment protocols. Nonetheless, these therapeutic strategies may still offer palliation and potential disease control in select cases, particularly when integrated into a comprehensive, multidisciplinary approach [11].

In recent years, immunotherapy with PD-1/PD-L1 inhibitors has gained prominence in the treatment of advanced squamous cell carcinoma, especially in cases where traditional systemic therapy has shown limited efficacy. In clinical trials (e.g., KEYNOTE-048), the use of pembrolizumab in patients with PD-L1-expressing head and neck SCC led to improved overall survival compared to platinum-based chemotherapy. In the case presented here, the decision to use immunotherapy was dictated by previous hematologic complications after chemotherapy and the potential benefits of immunomodulation of the tumor. Future research should focus on identifying predictive biomarkers of response to immunotherapy, such as PD-L1 expression, TMB (tumor mutational burden), or characterization of the tumor microenvironment. Advances in precision medicine are making it possible to better tailor therapies to an individual patient’s profile, which may improve treatment efficacy in cases such as the one described [20,22].

This case highlights the critical role of a multidisciplinary team in managing advanced malignancies with atypical metastatic patterns. The collaboration of oncologists, surgeons, radiologists, pathologists, and endocrinologists ensures a comprehensive evaluation and facilitates the formulation of individualized treatment plans. Such an approach is particularly vital in rare and complex cases where conventional treatment paradigms may not be applicable.

The presented case of metastatic squamous cell carcinoma of the larynx to the right adrenal gland exemplifies a rare and atypical pattern of distant dissemination. Advanced imaging modalities, particularly PET-CT, were instrumental in achieving an accurate diagnosis and staging of the disease. However, the inability to perform surgical resection due to local invasion highlighted the limitations of current therapeutic options.

This case underscores the importance of early detection and thorough staging of metastatic lesions, as timely intervention may improve outcomes in some patients. Furthermore, it highlights the urgent need for additional research into the efficacy of systemic therapies for such uncommon clinical scenarios. Investigating the role of emerging treatments, including novel immunotherapeutic agents and precision medicine approaches, could pave the way for improved management of rare metastatic patterns.

In conclusion, this case contributes to the growing body of literature emphasizing the importance of individualized patient care and interdisciplinary collaboration in oncology. It also serves as a reminder of the need for vigilance and adaptability in addressing the unique challenges posed by rare metastatic presentations, fostering a deeper understanding of this complex disease process.

## 6. Strengths and Limitations

The study has several strengths and limitations that are worth highlighting. One of its key strengths is the interdisciplinary approach, which involved a team of oncologists, surgeons, radiologists, and pathologists working together to ensure comprehensive evaluation and the creation of an individualized treatment plan. The use of advanced imaging techniques, particularly PET-CT, played a pivotal role in the precise localization of the metastatic lesion and assessment of its metabolic activity, which were crucial for both diagnosis and treatment planning. Additionally, the research employed a detailed and thorough methodology, including clinical and diagnostic evaluations such as laryngoscopic assessments, histopathological biopsy, and multimodal treatment strategies combining surgery, radiotherapy, and chemotherapy. Ethical considerations were also meticulously addressed, with the study adhering to rigorous standards that included informed consent, patient confidentiality, and compliance with institutional and international guidelines. Importantly, the study contributes to the medical literature by documenting a unique and rare case of metastatic squamous cell carcinoma of the larynx to the adrenal gland, providing valuable insights into uncommon metastatic patterns.

However, the study also has limitations. Its focus on a single case limits the generalizability of the findings to a broader population, as the results cannot be widely applied to other patients. The inability to surgically resect the adrenal tumor due to extensive invasion underscores significant challenges in the feasibility of current therapeutic options for such cases. Furthermore, there is a lack of standardized treatment protocols for rare metastatic patterns like the one described, which complicates the evaluation of the efficacy of alternative therapies. The research scope is somewhat restricted, as it does not delve deeply into emerging systemic therapies, such as immunotherapy or precision medicine, which might offer potential for similar cases. Lastly, the study provides limited follow-up data regarding systemic treatment outcomes, which prevents a thorough understanding of long-term disease control and survival rates for this type of metastasis. These factors highlight both the value and the constraints of this case study in advancing knowledge about rare metastatic presentations.

## 7. Conclusions

The presented case of squamous cell carcinoma of the larynx metastasizing to the adrenal gland highlights the complexities of diagnosing and managing rare metastatic patterns. This case underscores the importance of an interdisciplinary approach involving oncologists, surgeons, radiologists, and pathologists to ensure comprehensive evaluation and individualized treatment. Advanced imaging techniques, such as PET-CT, were instrumental in accurately identifying and assessing the metastatic lesion, showcasing their value in both diagnosis and treatment planning.

The inability to surgically resect the adrenal mass due to extensive invasion highlights the aggressive nature of such metastatic presentations and the limitations of current therapeutic options. This emphasizes the need for early detection and precise staging to improve potential outcomes. Additionally, the case demonstrates the critical role of distinguishing between functional and non-functional adrenal tumors in tailoring treatment strategies.

Despite the challenges, this case contributes valuable insights to the medical literature, providing a detailed example of an exceedingly rare metastatic presentation. It also underscores the urgent need for further research into the effectiveness of systemic therapies, including immunotherapy and precision medicine, in managing similar cases. This highlights a broader need for clinical studies and standardized protocols for guiding the treatment of rare and complex metastatic patterns.

Ultimately, the case reaffirms the necessity of vigilance, adaptability, and collaboration in oncology, particularly when dealing with atypical disease presentations. It serves as a reminder of the importance of combining advanced diagnostic tools, ethical clinical practices, and innovative therapeutic approaches to address the challenges of rare metastatic malignancies.

## Figures and Tables

**Table 1:** Case description and results.

**Case Overview**	66-year-old male with G3 SCC of the larynx and rare adrenal metastasis.
**Clinical Presentation**	Diagnosed in April 2020; significant locoregional tumor involvement; advanced SCC.
**Surgical Interventions**	Laryngectomy, selective cervical lymphadenectomy, and tracheostomy; no surgical complications; discharged in stable condition.
**Oncological Treatment**	Radiotherapy (70 Gy); chemotherapy interrupted due to leukopenia and thrombocytopenia; radiotherapy completed successfully.
**Follow-Up**	Imaging and biochemical evaluations; no local recurrence.
**Detection of Metastasis**	February 2023: Abdominal CT identified a 7 × 9 cm adrenal mass; PET-CT confirmed metabolic activity and retroperitoneal lymphadenopathy; hormonally inactive lesion.
**Surgical Evaluation**	March 2023: Laparotomy revealed unresectable adrenal mass; surgical biopsy confirmed SCC metastasis.
**Interpretation**	Advanced imaging pivotal for detection and evaluation; hormonal inactivity informed non-endocrine-focused therapy.
**Clinical Implications**	Highlights challenges of managing late-stage SCC with metastasis; emphasizes importance of early detection and interdisciplinary care.
**Summary**	Case underscores aggressive SCC nature; insights contribute to understanding rare metastatic presentations; Yin’s and PRISMA frameworks ensured systematic methodology.

## Data Availability

Data are contained within the article.
